# Assessing the Content Validity of the Revised Health of the Nation Outcome Scales (HoNOS 2018)

**DOI:** 10.3390/ijerph19169895

**Published:** 2022-08-11

**Authors:** Meredith G. Harris, Caley Tapp, Urska Arnautovska, Tim Coombs, Rosemary Dickson, Mick James, Jon Painter, Mark Smith, Angela Jury, Jennifer Lai, Philip M. Burgess

**Affiliations:** 1 School of Public Health, The University of Queensland, Public Health Building, Herston, QLD 4006, Australia; 2 Australian Mental Health Outcomes Classification Network (AMHOCN), The University of Queensland, Public Health Building, Herston, QLD 4006, Australia; 3 Australian Mental Health Outcomes Classification Network (AMHOCN), c/- HETI, Locked Bag 2030, St Leonards, NSW 1590, Australia; 4 Royal College of Psychiatrists, 21 Prescot Street, London E1 8BB, UK; 5 Department of Nursing and Midwifery, Collegiate Crescent, Sheffield Hallam University, Sheffield S10 2BP, UK; 6 Te Pou, Kakariki House, Waikato Mail Centre, 293 Grey St, Hamilton East P.O. Box 307, Hamilton 3240, New Zealand; 7 Te Pou, Millennium Centre, Phase II, Building B, Ground Floor, 600 Great South Road, Ellerslie, Auckland 1051, New Zealand

**Keywords:** content validity, measurement properties, mental health services, routine outcome measurement

## Abstract

The Health of the Nation Outcome Scales (HoNOS) comprises 12 scales that cover the kinds of problems that may be experienced by working-age adults in contact with specialised mental health services. Drawing on 20 years’ experience in clinical practice, a collaborative, international review of the HoNOS was undertaken and a revised measure (known as the HoNOS 2018) was published. In this study, 32 experts from Australia, England and New Zealand completed an anonymous web-based survey to assess the relevance, comprehensiveness and comprehensibility (aspects of content validity) of the HoNOS 2018. The experts rated 11 of the 12 HoNOS 2018 scales as ‘important’ or ‘very important’ for determining the overall clinical severity (item-level content validity index or I-CVI ≥ 0.75). Evaluations of the scales’ ability to capture change, comprehensiveness and comprehensibility were more variable, but generally positive. Experts’ comments provided further insights into this variability; for example, they noted that some scales combine multiple phenomena, which can result in ambiguity in item wording and assessment challenges. Results from this study suggest that the revisions have not altered the importance of the scales. Given the measure’s breadth of content, training remains important for ensuring rating fidelity. Inter-rater reliability and utility testing are indicated.

## 1. Introduction

In mental health services, routinely collected measures of clinical status and functioning are necessary tools for monitoring patient-level and service-level outcomes. The Health of the Nation Outcome Scales for working-age adults (HoNOS), developed in the mid-1990s, comprises 12 scales that cover the kinds of problems experienced by working-age adults in contact with specialised mental health services across the domains of behaviour, impairment, symptoms and social functioning (see Table 1). The clinician rates the severity of these problems on a 5-point scale (from 0 = no problem to 4 = severe to very severe problem), representing the maximum severity over the rating period, usually the previous two weeks. In assigning ratings, the clinician makes use of a glossary that provides summary rating instructions (general guidance that applies to all scales) as well as scale-specific guidance about what to include when making ratings, and descriptors that explain the meaning of each rating level (see [1]). The HoNOS forms part of a coordinated national approach to outcome measurement in England, Australia and New Zealand [2,3,4], with localised implementation in other countries including the Netherlands and Norway [5,6,7,8]. The measure is also used for research and funding purposes [2,3,9,10,11].

The HoNOS was developed following an extensive literature review and in consultation with experts from a range of disciplines [12]. It underwent four phases of testing and modification during a period of 3 years, which included large-scale field trials with 2706 patients from 25 sites [1]. After 20 years of use in clinical practice, a review of the HoNOS glossary was undertaken, led by the Royal College of Psychiatrists (the copyright holder) with representatives from Australia and New Zealand. The revision process is documented elsewhere [13]. Briefly, an advisory board was established, led by the Royal College of Psychiatrists and comprising members from England, Australia and New Zealand experienced in using the HoNOS for staff training, clinical practice, service monitoring and governance purposes. The board sought the opinions of clinicians in their networks regarding aspects of the HoNOS glossary that required refinement. Revisions were made in accordance with the criteria developed by the board. The revisions were intended to reduce ambiguity and inconsistency in the glossary, and to reflect changes in service delivery over time without changing the measure’s structure. Most of the 12 scales underwent a degree of revision. The nature of the changes varied across the scales but most often included changes to the scope of what to rate/include and/or the modification of examples given in the description for each rating level. A revised measure (known as the HoNOS 2018) was published in 2018 [13].

According to the COnsensus-based Standards for the selection of health Measurement INstruments (COSMIN) initiative, when a measure is modified, its measurement properties must be re-assessed. Content validity (whether the content of a measure adequately reflects the construct(s) of interest) is a priority because deficits in content validity can affect all other measurement properties [14,15]. The three aspects of content validity (relevance, comprehensiveness, and comprehensibility) should be assessed separately. Importantly, for multi-dimensional measures such as the HoNOS 2018, each scale should be considered separately [14]. Content validity studies of the original HoNOS found it to be generally appropriate, well designed and thorough. However, some concerns with individual scales have been identified, including the clarity of a rating level description and examples, and the scope of what is to be rated [16].

In its work providing advice and training to National Health Service (NHS) mental health providers in England, the Royal College of Psychiatrists is aware that whilst some services are using HoNOS 2018, others continue to use the original version of the HoNOS glossary. In Australia and New Zealand, evidence regarding the measurement properties and utility of HoNOS 2018 is needed to inform decisions about implementation. In this context, we aimed to assess the content validity of the 12 HoNOS 2018 scales.

## 2. Materials and Methods

### 2.1. Design and Participants

We designed a descriptive study in which HoNOS experts from Australia, England and New Zealand, (the only countries that had mandated the use of the HoNOS nationally at the time of this study) completed an anonymous, web-based survey. At least 10 participants were sought from each country with expertise in one or more of the following: making or supervising HoNOS ratings; psychometric or clinical effectiveness research involving the HoNOS; or using HoNOS ratings at a macro level (e.g., for training or monitoring service quality). Experts were identified through nominations from relevant national bodies and advisory groups (see Acknowledgements for details), bibliographic database searches, and the authors’ own professional networks. Members of the advisory board that revised the HoNOS were ineligible to participate.

Identified experts were emailed an invitation to participate in the survey. In some cases, an initial telephone contact was made to confirm contact details. The invitation email contained a link to the web-survey which, when selected, presented an information sheet and consent form. Upon providing informed consent, the participant entered the survey. Initial questions gathered basic information about participants’ professional backgrounds. The subsequent screens presented each scale of the HoNOS 2018 and corresponding content validity questions.

### 2.2. Survey

Six ‘core’ questions were developed, with input from all study authors, informed by best practice guidance and previous HoNOS content validity studies [13,14,15,17,18,19,20]. The questions were designed to measure the relevance, comprehensiveness and comprehensibility of each scale, taking into account the constructs being assessed, target population and context of use as necessary [14,15]. Draft questions were reviewed by members of Australia’s National Mental Health Information Development Expert Advisory Panel, and modifications were made in response to their feedback. The questions were:

1. How important is this scale for determining overall clinical severity for adult mental health service patients? (*relevance*)
2. How likely are repeat ratings on this scale to capture change in [scale-specific problems] during a period of mental health care? (*relevance*)
3. How well do the descriptors for each rating of 0–4 cover the range of [scale-specific problems] typically seen among adult mental health service patients? (*comprehensiveness*)
4. How helpful is the glossary for determining what to include when rating [scale-specific problems]? (*comprehensibility*)
5. How well do the descriptors for each rating of 0–4 correspond to the different levels of severity of [scale-specific problems]? (*comprehensibility*)
6. How consistent is the wording of the glossary with language used in contemporary mental health practice? (*comprehensibility*)

For each scale, in response to each question, experts indicated their opinion on a 4-point Likert scale (e.g., 1 = not important; 2 = somewhat important; 3 = important; 4 = very important). An additional open-ended question asked experts to provide further details if they rated 1 or 2 (a ‘negative’ rating) on any question for that scale. Upon completion of the survey, experts were invited to make final open-ended comments about the content of HoNOS 2018.

### 2.3. Ethics Approvals

Approval to conduct the study and to pool the data for analysis was obtained by each site—Australia (University of Queensland Medicine, Low & Negligible Risk Ethics Sub-Committee, 2019/HE002824; Research Ethics and Integrity, 2021/HE000113); England (Sheffield Hallam University Research Ethics Committee, ID ER21666298); and New Zealand (ethics review not required; Ministry of Health, Health and Disability Ethics Committees, 20/STH/109).

### 2.4. Analysis

Statistical analyses were conducted in Stata 16.0 (StataCorp, College Station, TX, USA). The proportion of experts who rated each scale positively on each core question was summarised using an item-level content validity index (I-CVI) [21,22]. To calculate the I-CVI, the sum of the number of ‘positive’ ratings (i.e., ratings of 3 or 4) was divided by the number of raters. This method of determining the I-CVI threshold takes the sample size into account, which addresses concerns about the inflation of agreement merely by chance [23,24]. At the 0.05 significance level and with at least 16 raters, an I-CVI value 0.75 or higher indicates ‘excellent’ content validity [21]. In addition, we calculated an average deviation (AD) index, which measures the dispersion of responses around the median [25]. At the 0.05 significance level and with at least 15 raters, for a four-point response scale, an AD index value lower than 0.68 indicates ‘excellent and statistically significant agreement’ [25]. Open-ended comments were analysed thematically [26] using NVivo 12 plus (QSR International, 2018). One team member analysed and coded the comments with the identification of categories. Themes were identified from these categories using a constant comparative method until new categories or themes ceased to emerge. Two other team members independently compared the themes to open-ended comments; revisions were made by consensus.

## 3. Results

A total of 43 experts (13 from Australia, 10 from England and 9 from New Zealand) were invited to participate, 32 (74%) completed the survey. Experts represented a mix of disciplines, although psychiatrists and nurses accounted for the majority. Most reported HoNOS expertise in at least 2 areas, the most common being clinical expertise. Collectively, they used HoNOS across a mix of settings. On average, they worked in mental health for 28 years and with the HoNOS for 15 years. More than half knew of HoNOS 2018, but few (9%) had used it in their work. (Table 2).

### 3.1. Experts’ Ratings of Each HoNOS 2018 Scale

The I-CVI values derived from experts’ ratings of the relevance, comprehensiveness and comprehensibility of each scale are shown in Table 3 and Table 4. Overall, the majority of ratings were ‘positive’, that is, 50% or more (i.e., I-CVI ≥ 0.5) of experts gave positive ratings on all but one of the 72 core questions, and 70% (i.e., I-CVI ≥ 0.7) did so on nearly 70% of core questions.

Eleven of the twelve scales met the *a priori* criterion for excellent content validity (I-CVI ≥ 0.75) on the question assessing importance for determining the overall clinical significance (an indicator of relevance). On the question assessing the helpfulness of the glossary for determining what to rate and/or include (*comprehensibility*), nine scales met the criterion. On the questions assessing the likelihood of capturing change during a period of mental health care (*relevance*), the correspondence between descriptors and levels of severity (*comprehensibility*) and the consistency of wording with contemporary mental health practice (*comprehensibility*), six scales met the criterion. On the question assessing the coverage of problems typically seen among adult mental health service consumers/patients (*comprehensiveness*), five scales met the criterion.

Three scales met the criterion on all questions. These were: Scale 6 (Problems associated with hallucinations and/or delusions), Scale 7 (Problems with depressed mood) and Scale 9 (Problems with relationships). Two scales—Scale 4 (Cognitive problems) and Scale 10 (Problems with activities of daily living)—met the criterion on all except the ‘likelihood of capturing change’ question. Conversely, three scales met the criterion on only 1 or 2 questions: Scale 3 (Problem drinking or drug-taking), Scale 5 (Physical illness or disability problems) and Scale 11 (Problems with housing and living conditions). 

All AD index values were below the critical value of 0.68, indicating acceptable and clinically significant agreement between experts.

### 3.2. Experts’ Concerns

Analysis of experts’ reasons for their ‘negative’ ratings revealed six themes related to comprehensibility, two to relevance and one to comprehensiveness. An additional theme indicated possible areas for focus in training. The themes are summarised below, with illustrative quotations.

### 3.3. Themes about Comprehensibility

#### 3.3.1. Too Many Phenomena

A recurring concern was that some scales combine several different phenomena.

“Overactive behaviour is not the same as aggressive behaviour and both cannot be sensibly combined into a single rating”.

(Scale 1. Overactive or aggressive or disruptive or agitated behaviour)

“Conflating iatrogenic or highly transitory states with long-term and enduring disability is problematic”.

(Scale 5. Physical illness or disability problems)

#### 3.3.2. Ambiguity

Although the HoNOS review project aimed to reduce ambiguity in the glossary, experts continued to identify ambiguity in terminology and rating instructions.

“‘D’ is labelled ‘Reactions to stressful events and trauma.’ […] It is not clear whether only acute stressors and traumas are to be coded (and if so how recent the event might have been). This is ambiguous”.

(Scale 8. Other mental and behavioural problems)

Some commented that ambiguity can arise when a rating requires a comparison to contextual norms.

“What does “Excessive” mean. More than the rater? This needs better anchors. Would any Ice use be excessive?”.

(Scale 3. Problem drinking or drug-taking)

#### 3.3.3. Need for More Description or Examples

Given concerns about rating too many phenomena and ambiguity, it is not surprising that there were calls for more descriptions or examples to be added to the glossary.

“Descriptors would be far more useful if they simply gave examples of the types of acts one would expect at each rating level. Examples are only given for a minority of the descriptors”.

(Scale 1. Overactive or aggressive or disruptive or agitated behaviour)

#### 3.3.4. Assessment Challenges 

Given the variety of phenomena to be considered, discriminating between these phenomena across and within scales for the purposes of rating can be an assessment challenge.

“Making a distinction between behavioural aspects of drug/alcohol use (rated here) and aggressive/destructive behaviour rated in Scale 1 can be problematic”.

(Scale 3. Problem drinking or drug-taking)

Other assessment challenges were related to the assessment context.

“The glossary seems entirely focussed on community patients and does not describe how to approach this scale if a patient is being treated in a residential setting e.g., inpatient ward in hospital”.

(Scale 11. Problems with housing and living conditions)

#### 3.3.5. Lack of Fit with Clinical Thinking

Although the glossary’s instructions regarding what to rate/include were generally well understood, experts identified a lack of fit with their thinking about certain clinical problems.

“Staff may continue to think in terms of depression rather than depressed mood irrespective of how it is worded”.

(Scale 7. Problems with depressed mood)

“The difficulty I have with this catch-all item is that it contains the most common presentations […] in one question. In an ideal world, there would be an optional drop-box that permits these to be rated separately”.

(Scale 8. Other mental and behavioural problems)

#### 3.3.6. Problems with Language

There was feedback that some wording used in the glossary does not align with clinical language or constructs.

“Occupation is a bit narrow (both the language as well as the construct)”.

(Scale 12. Problems with occupation and activities)

There was also feedback that some wording could be viewed as pejorative:

““Passive” is not an ideal term-requires a judgement which is not easily made and is potentially pejorative”.

(Scale 2. Non-accidental self-injury)

### 3.4. Themes about Relevance

#### 3.4.1. Importance

Only one scale was the subject of comments about importance.

“Housing is not part of the clinical formulation but part of the contextual background”.

(Scale 11. Problems with housing and living conditions)

“Not sure of the value of rating inpatient setting”.

(Scale 11. Problems with housing and living conditions)

#### 3.4.2. Capturing Change

Some scales were perceived as lacking sensitivity to certain patterns of change that may take place over an episode of care.

“Difficult to capture patients with emotionally unstable personality disorder who can have daily ideas suicide & frequent self-harm attempts”.

(Scale 2. Non-accidental self-injury)

“May be less likely to pick up change in capacity in an episode of care compared with most other scales, as there is often a lag in these resuming as clinical state improves”.

(Scale 10. Problems with activities of daily living)

Some commented that the cause of the behaviour is an important consideration.

“Depending on the cause of the problem, change may be slow/absent/minor”.

(Scale 4. Cognitive problems)

### 3.5. Themes about Comprehensiveness

#### Coverage

For a few scales, experts suggested additions to the examples given in the rating level descriptions.

“Self-harming behaviour, e.g., cutting, skin picking/hair pulling/ head banging/burning (cigarette burns) without suicidal thoughts especially when these present as longer term chronic mal-adaptive behaviour aimed at self-management of emotions are not included in descriptors”.

(Scale 2. Non-accidental self-injury)

“Scale appears to be useful for the most seriously impaired, but not fine grained enough, doesn’t include wider range of roles-parenting, caregiving, training, cultural”.

(Scale 12. Problems with occupation and activities)

### 3.6. Areas for Focus in Training

Some comments pointed to areas of clarification that could be a focus for training.

“Instructions regarding the need to incorporate cultural and contextual factors into ratings?-minimal guidance is provided as to how such factors may need to be considered, hopefully this would be addressed in any training package”.

(Summary rating instructions)

“Again, it would be good to clarify if this is to be rated from the clinician’s perspective or, more consistent with a recovery approach, the patient’s perspective?”

(Scale 12. Problems with occupation and activities)

“Is this item attempting to capture the availability of occupation/activity or the patient’s ability and or motivation to engage in activity?”

(Scale 12. Problems with occupation and activities)

### 3.7. Experts’ Final Comments

Although experts were not asked in the survey to directly compare the original HoNOS to the HoNOS, several said they favoured the HoNOS 2018.

“The revisions in HoNOS 2018 brings more clarity to the scales within HoNOS which is likely to improve the overall validity and reliability of the scale. The revisions are well thought through as they maintain the integrity of the original measure.”

Others commented that, irrespective of the revisions, the value of the measure is limited if it is not used to guide clinical decision making and care.

“Key issue is clinicians using these rating scales to guide care provision. This will drive up accuracy & consistency. Unfortunately, scales are seen as performance measure to be completed not one of range of tools to help with assessment of patient’s needs.”

## 4. Discussion

To our knowledge, this is the first empirical study of HoNOS 2018. A key finding was the strong consensus between experts that HoNOS 2018 scales are important for determining overall clinical severity. This is consistent with a study examining the importance of the original HoNOS scales [20] and provides some reassurance that the glossary revisions have not altered the measure’s relevance, a core aspect of content validity. Only Scale 11 (Problems with housing and living conditions) had an I-CVI below the 0.75 threshold for excellent content validity (0.71), likely reflecting experts’ concerns about its relevance in inpatient settings and its less direct focus on patient need. 

Evaluations of each scale’s ability to capture change, comprehensiveness and comprehensibility were more variable, although most experts rated most scales positively. Thematic analysis revealed possible explanations for this variability. For example, a theme related to comprehensibility was that some scales combine multiple phenomena, which may result in ambiguity in item wording and assessment challenges. Indeed, scales that consistently met the criterion for excellent content validity—for example, Scale 7 (Problems with depressed mood)—tended to focus on a single phenomenon or a relatively narrower range of phenomena. Conversely, the scales that describe behavioural problems—Scale 1 (Overactive or aggressive or disruptive or agitated behaviour), Scale 2 (Non-accidental self-injury) and Scale 3 (Problem drinking or drug-taking)—were frequently noted as entailing multiple phenomena and being insufficiently illustrated with examples, making it challenging to determine a severity rating. These scales had lower I-CVIs on the survey question about correspondence between descriptors and severity levels. 

Another theme related to comprehensibility was a perceived lack of fit between the intention of the ratings and usual clinical thinking for certain problems. For example, for Scale 5 (Physical illness or disability problems), some experts perceived the focus on activity restrictions as too narrow and wanted an opportunity to include issues related to chronic physical health problems (e.g., risk of future adverse consequences). This scale had relatively lower I-CVIs on all comprehensibility questions. This concern has not been raised in content validity studies of the original HoNOS. For Scale 8 (Other mental and behavioural problems), several experts expressed a desire to rate multiple problems; a view that has previously been reported [13,27,28], but was considered out of scope for the revision because it would require a structural change to the measure [13]. These concerns may reflect heightened recognition in recent years of the prevalence and outcomes of multi-comorbidity among people with severe mental illness [29,30]. 

With respect to comprehensiveness, experts suggested some specific additions to the rating level descriptions for some scales (e.g., examples of specific types of self-harm in Scale 2); these have not been raised in previous studies. Conversely, a lack of opportunities to rate thought disorder, elated mood and post-traumatic stress disorder have been raised in previous studies [13,31]. These gaps were addressed in the revision by changing the scope of what to rate and include in Scale 4 (Cognitive problems) and Scale 8 (Other mental and behavioural problems). These concerns were not raised in the current study, suggesting they were adequately addressed by the revision.

## 5. Implications

In services already using HoNOS 2018, findings from this study could help to refine training and support materials. For example, although the HoNOS 2018 includes additional guidance about incorporating cultural and contextual factors into ratings [13], some experts called for further explanation and examples. These comments underscore the importance of cultural competence as a broader framework to guide clinical practice, including HoNOS/HoNOS 2018 ratings. Training provides an opportunity to address identified assessment challenges—for example, distinguishing when to rate patient motivation versus opportunities in their environment, which is a difficult task that may require additional support materials [32]. Training also provides an opportunity to reinforce that, although HoNOS 2018 permits a summary of assessments across a broad range of important constructs, it does not replace clinical judgement or preclude other clinical issues being documented.

The study findings may help inform decisions about HoNOS 2018 implementation in services where this is being considered. However, evidence regarding inter-rater reliability and other measurement properties [13], utility, infrastructure costs, and training implications may also need to be considered. Findings may also assist in interpreting results from future studies of the HoNOS 2018’s measurement properties [14] and could inform future revisions of the content of the scales. 

Although several experts said they expected the revisions to result in improved reliability, validity and sensitivity to change, others perceived the lack of clinical utility to be of greater concern. This concern may reflect broader views about the value of the routine outcome measurement [33,34,35,36], as much as specific HoNOS or HoNOS 2018 limitations. Regardless, there remains a gap in knowledge about how the HoNOS is used or perceived as useful by clinicians [37]. Future studies could involve asking raters who used the HoNOS 2018 in their day-to-day practice, how useful it is for different purposes (e.g., as a tool to inform treatment planning, as a tool to monitor clinical change) and whether they find it more, less or equally useful compared to the original HoNOS. 

In this study, we focused on clinicians’ perspectives of content validity, which is appropriate for a clinician-rated measure [38], but future studies exploring patient perspectives could be a useful complement.

## 6. Strengths and Limitations

The three participating countries have heavily invested in implementing the HoNOS in their national mental health outcome measurement efforts but have different service systems and different HoNOS training materials and delivery systems. This supports the ‘real-world’ applicability of the findings. The sample size is considered adequate (i.e., ≥30) for a quantitative content validity study, and we included a qualitative component to facilitate a deeper understanding of the experts’ perspectives [15].

Some limitations should be noted. First, various biases may have occurred. Approximately one-quarter of invited experts did not complete the survey. We do not know whether non-completers may have held different views to completers, however, the survey responses revealed a mix of positive and negative views among participating experts. There may have been selection biases, however, the use of multiple strategies to identify experts may have helped mitigate these. Second, every effort was made to identify suitable experts who met the pre-determined definition of having HoNOS expertise, but we did not verify their expertise against stringent criteria [39,40]. However, experts reported that they had worked with the HoNOS for many years, and most reported expertise with HoNOS ratings coupled with research or service-related expertise. Third, to reduce respondent burden, we only asked experts to elaborate on their ‘negative’ ratings. This means that the qualitative results emphasise concerns and should be considered alongside the quantitative results for a balanced interpretation.

## 7. Conclusions

After 20 years of use in clinical practice, the HoNOS glossary was revised, resulting in an updated measure known as the HoNOS 2018. In this study, there was strong consensus among experts that the HoNOS 2018 scales are relevant for determining the clinical severity of adults in contact with specialised mental health services, suggesting that the revision of the measure has not altered this core aspect of its content validity. For the most part, experts considered the HoNOS 2018 scales to be adequate in terms of capturing change, comprehensiveness and comprehensibility. Given the measure’s breadth of content, training and support materials remain necessary to address areas of ambiguity and encourage rating fidelity. Findings are sufficiently encouraging to warrant further exploration of the utility, inter-rater reliability and other measurement properties of the HoNOS 2018.

## Figures and Tables

**Table 1:** The HoNOS/HoNOS 2018 scales.

Scale Titles	Range of Scale Scores ^a^
1. Overactive or aggressive or disruptive or agitated behaviour ^b^	0–4
2. Non-accidental self-injury	0–4
3. Problem drinking or drug-taking	0–4
4. Cognitive problems	0–4
5. Physical illness or disability problems	0–4
6. Problems associated with hallucinations and/or delusions ^c^	0–4
7. Problems with depressed mood	0–4
8. Other mental and behavioural problems	0–4
9. Problems with relationships	0–4
10. Problems with activities of daily living	0–4
11. Problems with housing and living conditions ^d^	0–4
12. Problems with occupation and activities	0–4

HoNOS, Health of the Nation Outcome Scales. ^a^ Scales are rated on a 5-point scale: 0 = no problem; 1 = minor problem requiring no action; 2 = mild problem but definitely present; 3 = moderately severe problem; 4 = severe to very severe problem. ^b^ In the original HoNOS, the title for Scale 1 is ‘Overactive, aggressive, disruptive or agitated behaviour’. ^c^ In the original HoNOS, the title for Scale 6 is ‘Problems associated with hallucinations and delusions’. ^d^ In the original HoNOS, the title for Scale 11 is ‘Problems with living conditions’.

**Table 2:** Characteristics of experts who completed the survey (N = 32).

	*n*	%
**Main professional background ^a^**		
Nurse	7	23
Psychologist	3	10
Psychiatrist	16	52
Other ^b^	5	16
**Expertise in working with HoNOS ^c^**		
Rating HoNOS or reviewing HoNOS ratings made by others	27	84
Research in the measurement properties of the HoNOS and/or measuring clinical effectiveness	20	63
HoNOS staff training and/or using HoNOS results at a macro level	20	63
**Mental health settings worked with HoNOS ^c^**		
Inpatient	22	69
Residential ^d^	7	22
Community	30	94
Other, non-clinical setting	4	13
**Aware of HoNOS 2018 prior to survey**		
No, I was not aware of the HoNOS 2018 at all	12	38
Yes, I was aware of the HoNOS 2018, but have not used it in my work	16	50
Yes, I have used the HoNOS 2018 in my work	3	9
Other	1 ^e^	3
	**M (SD)**	**Range**
**Years worked in mental health ^f^**	28 (9)	10–43
**Years worked with the HoNOS**	15 (5)	5–28

HoNOS, Health of the Nation Outcome Scales. M, mean. SD, standard deviation. ^a^ Missing data for one respondent (*n* = 31). ^b^ Included clinical psychologist, social worker, clinical epidemiologist, consumer/carer/family advisor/leader. ^c^ Categories not mutually exclusive. ^d^ ‘Residential’ category included only in the Australian version of the survey. ^e^ “I have not seen the revised HoNOS 2018”. ^f^ Missing data for two respondents (*n* = 30).

**Table 3:** Experts’ ratings of the content validity of the HoNOS 2018 scales: relevance and comprehensiveness.

	Relevance								Comprehensiveness			
	How Important Is This Scale for Determining Overall Clinical Severity for Adult Mental Health Service Consumers/Patients? ^a^				How Likely Are Repeat Ratings on This Scale to Capture Change in [Scale-Specific Problems] during a Period of Mental Health Care?				How Well Do the Descriptors for Each Rating of 0–4 Cover the Range of [Scale-Specific Problems] Typically Seen among Adult Mental Health Service Consumers/Patients? ^a,b^			
HoNOS 2018 Scale	*n*	Range	I-CVI	AD Index	*n*	Range	I-CVI	AD Index	*n*	Range	I-CVI	AD Index
Scale 1. Overactive or aggressive or disruptive or agitated behaviour	31	2–4	**0.81**	0.48	30	1–4	0.67	0.53	32	1–4	0.72	0.50
Scale 2. Non-accidental self-injury	31	2–4	**0.90**	0.55	32	2–4	0.66	0.47	31	1–4	0.65	0.48
Scale 3. Problem drinking or drug-taking	32	2–4	**0.94**	0.38	31	1–4	0.55	0.61	31	1–4	0.65	0.61
Scale 4. Cognitive problems	32	2–4	**0.91**	0.41	32	1–4	0.66	0.63	32	1–4	**0.88**	0.31
Scale 5. Physical illness or disability problems	30	1–4	**0.77**	0.40	32	1–4	0.56	0.66	31	1–4	0.71	0.55
Scale 6. Problems associated with hallucinations and /or delusions	31	2–4	**0.97**	0.52	32	2–4	**0.88**	0.50	32	1–4	**0.81**	0.56
Scale 7. Problems with depressed mood	32	2–4	**0.97**	0.50	32	2–4	**0.81**	0.50	32	1–4	**0.88**	0.41
Scale 8. Other mental and behavioural problems	32	2–4	**0.88**	0.44	32	1–4	0.69	0.44	32	1–4	0.69	0.50
Scale 9. Problems with relationships	31	2–4	**0.87**	0.39	32	1–4	**0.81**	0.31	32	1–4	**0.78**	0.34
Scale 10. Problems with activities of daily living	32	2–4	**0.91**	0.25	31	1–4	0.74	0.39	31	1–4	**0.81**	0.39
Scale 11. Problems with housing and living conditions	31	1–4	0.71	0.45	29	1–4	**0.79**	0.45	32	1–4	0.66	0.50
Scale 12. Problems with occupation and activities	30	1–4	**0.77**	0.47	32	1–4	**0.75**	0.34	32	1–4	0.66	0.56

HoNOS, Health of the Nation Outcome Scales. I-CVI, item-level content validity index. AD, average deviation. *n*, number. Bold I-CVI values meet criteria for excellent content validity (i.e., I-CVI ≥ 0.75). ^a^ Australian and New Zealand versions of the survey used ‘consumer’; the England version used ‘patient’. ^b^ To fit the wording of Scale 8, the equivalent question for Scale 8 was: How well do problems A-O cover the range of other mental and behavioural problems typically seen among adult mental health service patients?

**Table 4:** Experts’ ratings of the content validity of the HoNOS 2018 scale: comprehensibility.

	Comprehensibility											
	How Helpful Is the Glossary for Determining What to Include When Rating [Scale-Specific Problems]? ^a,b^				How Well Do the Descriptors for Each Rating of 0–4 Correspond to the Different Levels of Severity of [Scale-Specific Problems]?				How Consistent is the Wording of the Glossary with Language Used in Contemporary Mental Health Practice?			
HoNOS 2018 Scale	*n*	Range	I-CVI	AD Index	*n*	Range	I-CVI	AD Index	*n*	Range	I-CVI	AD Index
Scale 1. Overactive or aggressive or disruptive or agitated behaviour	32	2–4	**0.78**	0.44	32	1–4	0.59	0.50	30	1–4	**0.80**	0.33
Scale 2. Non-accidental self-injury	31	2–4	**0.81**	0.35	32	1–4	0.50	0.75	32	1–4	**0.75**	0.38
Scale 3. Problem drinking or drug-taking	32	1–4	**0.75**	0.50	31	1–4	0.65	0.58	32	1–4	0.69	0.53
Scale 4. Cognitive problems	31	2–4	**0.84**	0.42	30	1–4	**0.87**	0.27	30	1–4	**0.83**	0.27
Scale 5. Physical illness or disability problems	22 ^c^	1–4	0.45	0.64	32	1–4	0.50	0.69	30	1–4	0.67	0.50
Scale 6. Problems associated with hallucinations and /or delusions	32	2–4	**0.88**	0.44	32	2–4	**0.88**	0.41	32	2–4	**0.88**	0.28
Scale 7. Problems with depressed mood	32	1–4	**0.78**	0.66	31	2–4	**0.81**	0.45	32	1–4	**0.81**	0.34
Scale 8. Other mental and behavioural problems	32	2–4	**0.78**	0.47	31	2–4	0.68	0.42	32	1–3	**0.81**	0.22
Scale 9. Problems with relationships	32	2–4	**0.75**	0.31	32	2–4	**0.78**	0.38	31	1–4	**0.77**	0.35
Scale 10. Problems with activities of daily living	32	2–4	**0.88**	0.28	32	1–4	**0.91**	0.22	32	2–4	**0.88**	0.22
Scale 11. Problems with housing and living conditions	32	1–4	0.69	0.50	31	1–4	0.74	0.42	31	1–4	**0.77**	0.29
Scale 12. Problems with occupation and activities	32	1–4	0.59	0.53	32	1–4	**0.75**	0.38	32	1–4	0.69	0.50

HoNOS, Health of the Nation Outcome Scales. I-CVI, item-level content validity index. AD, average deviation. *n*, number. Bold I-CVI values meet criteria for excellent content validity (i.e., I-CVI ≥ 0.75). ^a^ Question text differed across scales; depending on the glossary, “what to rate and include” or “what to rate and consider” was substituted for the phrase “what to include”. ^b^ To fit the wording of Scale 8, the equivalent question for Scale 8 was: How helpful is the glossary for determining which other mental and behavioural problem to rate on this scale? ^c^ This question was inadvertently omitted from the England survey.

## Data Availability

The datasets generated and analysed for the current study are not available.
